# Association Between Maternal Smoking, Isolated Proteinuria During Pregnancy and Preterm Birth: A Finnish Registry Analysis

**DOI:** 10.1016/j.ekir.2024.10.025

**Published:** 2024-10-28

**Authors:** Mikael O. Ekblad, Mika Gissler, Päivi E. Korhonen

**Affiliations:** 1 Department of General Practice, Institute of Clinical Medicine, University of Turku and Turku University Hospital, Turku, Finland; 2 Department of Knowledge Brokers, Helsinki, THL Finnish Institute for Health and Welfare, Finland; 3 Research Centre for Child Psychiatry, University of Turku, Turku, Finland; 4 Department of Molecular Medicine and Surgery, Karolinska Institute, Stockholm, Sweden; 5 Region Stockholm, Academic Primary Health Care Centre, Stockholm, Sweden

**Keywords:** pregnancy, prenatal, tobacco

## Abstract

**Introduction:**

Smoking during pregnancy (SDP) seems to paradoxically decrease the likelihood of preeclampsia. We aimed to investigate the association between smoking and isolated proteinuria during pregnancy. In addition, we investigated the associations and potential interaction between smoking and proteinuria on the risk for preterm birth.

**Methods:**

The study included all women with singleton pregnancies (*N* = 791,183) in Finland during the years 2006 to 2018, excluding those with previous kidney diseases, gestational hypertension or diabetes, or preeclampsia. Information on smoking and background factors were derived from the Finnish Medical Birth Register. Smoking was categorized as no smoking, quit in the first trimester, or continued smoking thereafter. Information on isolated proteinuria at any time of pregnancy was derived from the Finnish Hospital Discharge Register and the Finnish Medical Birth Register with an International Classification of Diseases (ICD) (Tenth Revision [ICD-10]) code O12, excluding gestational edema (O12.0). Logistic and linear regression models were used to estimate the associations.

**Results:**

Of the participants, 14.6% were smokers, of which 36.9% quit smoking; 2534 (0.3%) had a diagnosis of isolated proteinuria. Those who quit smoking (odds ratio [OR] = 1.31, 95% confidence interval [CI] = 1.14–1.52) and those who continued smoking (OR = 1.29, 95% CI=1.15-1.46) were associated with having a diagnosis of isolated proteinuria. Isolated proteinuria (OR = 1.24, 95% CI = 1.03–1.49) and those who continued smoking (OR = 1.45, 95% CI = 1.40–1.50) were associated with preterm birth. The interaction of smoking and isolated proteinuria with preterm birth lacked statistical significance.

**Conclusion:**

We found evidence of an association between smoking and the diagnosis of isolated proteinuria. Furthermore, smoking and a diagnosis of isolated proteinuria were both associated with a higher risk for preterm birth.

## Graphical abstract

**Figure:**
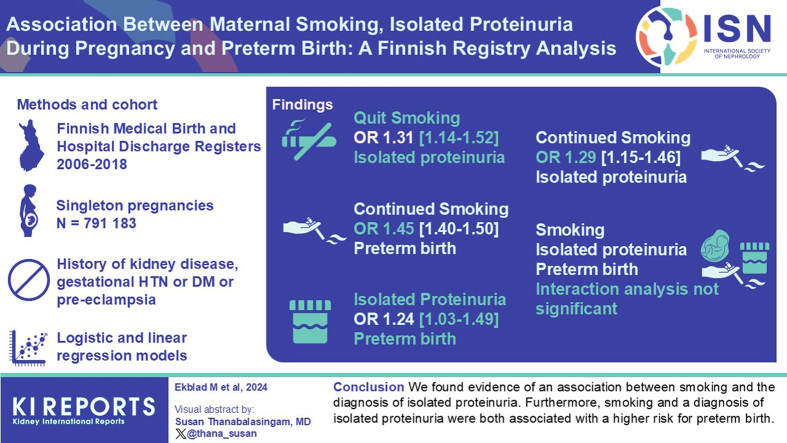

Maternal SDP has a wide range of adverse effects on maternal health such as increased risk for premature delivery and the need for hospital treatment during pregnancy[;^1^, ^2^, ^3^ as well as on fetal outcomes such as lower birth weight, head circumference, and brain development of the newborn.^4^^,^^5^ SDP also has long-lasting adverse effects on the child's health.^6^^,^^7^ SDP has been found to have a dose-relationship with many outcomes.^4^^,^^7^

Based on several studies, maternal SDP seems to paradoxically decrease the likelihood of preeclampsia and gestational hypertension.^8^, ^9^, ^10^ It has been speculated that tobacco combustion products, especially carbon monoxide, are the probable protective ingredients against preeclampsia and gestational hypertension in cigarette smoke rather than nicotine.^8^^,^^9^ For example, pregnant women who used snuff during pregnancy had a similar or even slightly higher risk for preeclampsia compared to women who did not use nicotine products, whereas smokers had significantly lower risk for preeclampsia.^9^

The main diagnostic criteria for preeclampsia have, until recent years, included the presence of elevated blood pressure and proteinuria.^11^ The question remains whether SDP is associated with isolated proteinuria during pregnancy even with the lower risk for preeclampsia and gestational hypertension. Until recent years, mild isolated proteinuria has been considered to be physiological, thus the long-term consequences have not been studied. A study by Kreepala *et al.*^12^ found that even mild proteinuria might be an early biomarker for renal pathology rather than being physiological. During pregnancy, women with isolated proteinuria have been found to be more likely to progress to preeclampsia than women with isolated hypertension.^13^ Furthermore, Ghamrawi *et al.*^14^ have speculated that the occurrence of proteinuria during pregnancy might be the first manifestation of underlying subclinical kidney disease.

We aimed to investigate the association between SDP and isolated proteinuria during pregnancy by using a population-based longitudinal register data. In addition, we aimed to investigate the association of and potential interaction between SDP and isolated proteinuria with the duration of pregnancy and the risk of preterm birth.

## Methods

### Data Sources

This study was based on data from the Finnish Medical Birth Register and the Finnish Hospital Discharge Register. The Finnish Institute for Health and Welfare, which is the current register keeper, performed the ethical evaluation and granted the permission to use their confidential health register data, as required by national data-protection legislation. Pregnant mothers’ unique personal identification numbers were used to combine all register data. The data linkages were performed by the statistical authorities and only unidentifiable data were provided for the researchers outside the Finnish Institute for Health and Welfare.

The Medical Birth Register includes all live births and stillbirths of fetuses with a gestational age of ≥22 weeks or with a birthweight of ≥500 g. The register keeper collects the data from all delivery hospitals and, in the case of home births, from the assisting health care personnel. The register includes information on the mother's and the child's identification numbers; maternal background, health care, and interventions during pregnancy and delivery; and the newborn's health status until aged 7 days. The Medical Birth Register is considered to be a complete record of all births and newborns in Finland. Most of the register content corresponds well or satisfactorily with hospital record data according to 2 data quality studies.^15^^,^^16^

The Hospital Discharge Register includes information on all episodes of inpatient care (including all hospitalizations requiring an overnight stay) in public and private hospitals since 1967 and outpatient visits in public hospitals since 1998. The register contains information on the patient's background, hospitalization period, procedures, and the main diagnosis and secondary diagnoses by ICD code (Eight Revision [ICD-8] in 1969–1986, Ninth Revision [ICD-9] in 1987–1995, and ICD-10 since 1996). A systematic review showed that the completeness and accuracy of the register range from satisfactory to very good.^17^

### Study Sample

The study population consisted of all pregnant women with singleton pregnancies (*N* = 835,551) in Finland between 2006 and 2018. The follow-up began in 2006 because this was the year all birth hospitals started providing information on maternal height and weight. ICD-10 classification was used during the entire study period. Women with a prepregnancy diagnosis of chronic hypertension (ICD-10 codes: O10 and O11) were excluded (*n* = 7657) as was done in our previous study.^8^ In addition, women with a diagnosis of preeclampsia (ICD-10 codes: 014 and O15, *n* = 5955), prepregnancy kidney diseases (ICD-10 codes: N00–N29, *n* = 898) and gestational diabetes (ICD-10 code: O24, *n* = 6,047) were excluded. The information on SDP was missing from 23,811 singleton pregnancies (2.9%), which were excluded from the statistical analysis. The final study population consisted of 791,183 healthy pregnant women (94.7%) of all singleton pregnancies during the study period.

Information on SDP, duration of pregnancy (gestational weeks + days), and other background factors (maternal age, parity, and prepregnancy body mass index [BMI]) were derived from the Finnish Medical Birth Register. Midwives collected smoking information from the mothers during antenatal care. SDP was categorized into 3 groups: (i) participants who did not smoke during pregnancy (no smoking), (ii) participants who quit smoking during the first trimester (quit smoking), and (iii) participants who continued smoking after the first trimester (continued smoking).

### Outcome Diagnoses

In Finland, expectant mothers normally meet with a nurse or/and doctor 11 to 15 times during pregnancy and 99% of women participate in the maternity clinic follow-ups. Proteinuria during pregnancy is screened repeatedly before every visit by using the urine strip test. If the strip test is positive, more detailed examinations of proteinuria are performed according to the Finnish Current Care Guidelines for gestational hypertension and preeclampsia.^11^ Information on isolated proteinuria was derived from the Hospital Discharge Register and the Medical Birth Register. ICD-10 diagnostic codes O10 to O15 regarding edema; proteinuria; and hypertensive disorders in pregnancy, childbirth, and puerperium for each pregnancy were obtained from 2006 through 2018. In this study, isolated proteinuria was defined by ICD-10 code group O12 gestational (pregnancy-induced) edema and proteinuria without hypertension, excluding code O12.0 gestational edema.

### Statistics

First, logistic regression models were used to estimate the association between SDP and isolated proteinuria. In the unadjusted model, isolated proteinuria was added as the independent variable and SDP as the dependent categorical variable. In the adjusted model, we added maternal age and parity as continuous covariates and prepregnancy BMI as a categorical covariate into the model.

Second, we studied the association of SDP, proteinuria, and other covariates with the risk for preterm birth (below 37 weeks of gestation, yes/no) by using logistic regression model, and duration of pregnancy (in days, excluding early preterm births with gestational age < 32 weeks) by using linear regression model. These models can be thought of as an approximation of the standard comparison seen in the literature. Finally, SDP and proteinuria interaction was added to both previous models to explore whether maternal proteinuria modifies the association between SDP and preterm birth/duration of pregnancy similar to our previous study.^8^ Normal distribution assumption was checked from studentized residuals. Sensitivity analyses were performed similarly by using linear regression model on the association of SDP and proteinuria with the duration of pregnancy (in days) for the whole study population without excluding early preterm births. Gestational age 40 weeks + 0 days = 280 days was used as a reference.

The data analysis was performed using commercially available software (SAS, version 9.4; SAS Institute Inc., Cary, NC). Differences in the results were evaluated using 95% CIs and *P*-values. CIs which do not overlap with the null values and *P*-values < 0.05 were considered to be significant.

## Results

The characteristics of the study population (*N* = 791,183) by maternal SDP is presented in Table 1. A total of 14.6% were smokers during pregnancy of which 36.9% quit smoking during the first trimester of pregnancy. Of the participants, 2534 (0.3%) had received a diagnosis of isolated proteinuria after excluding women with unknown smoking status, diagnosis of preeclampsia, prepregnancy chronic hypertension, kidney diseases, or gestational diabetes.

**Table 1:** Characteristics of the study population by smoking information

Characteristics	No smoking, *n* (%)	Quit smoking, *n* (%)	Continued smoking, *n* (%)	Missing information of smoking, *n* (%)	*P*-value
All	672,622 (82.53)	43,690 (5.36)	74,871 (9.19)	23,811 (2.92)	
Maternal age (yrs)					
< 20	8712 (1.30)	2,360 (5.40)	6128 (8.18)	566 (2.38)	<0.001
20–34	524,928 (78.04)	36,747 (84.11)	59,408 (79.35)	18,138 (76.17)	
35–39	112,830 (16.77)	3854 (8.82)	7425 (9.92)	4074 (17.11)	
≥ 40	26,152 (3.89)	729 (1.67)	1910 (2.55)	1033 (4.34)	
Parity					
0	270,498 (40.22)	25,735 (58.90)	33,245 (44.40)	7844 (32.94)	<0.001
1	235,065 (34.95)	11,091 (25.39)	21,658 (28.93)	8447 (35.48)	
2–3	133,351 (19.83)	6068 (13.89)	16,529 (22.08)	5639 (29.56)	
≥ 4	33,545 (4.99)	789 (1.81)	3423 (4.57)	1542 (6.48)	
Unknown	163 (0.02)	7 (0.02)	16 (0.02)	339 (1.42)	
Body mass index (kg/m^2^)					
< 20	87,120 (12.95)	5,969 (13.66)	11,653 (15.56)	2297 (9.65)	<0.001
20.0–24.9	345,454 (51.36)	20,355 (46.59)	31,396 (41.93)	8417 (35.35)	
25.0–29.9	140,506 (20.89)	10,189 (23.32)	16,574 (22.14)	3799 (15.95)	
30.0–34.9	49,945 (7.43)	4205 (9.62)	7630 (10.19)	1493 (6.27)	
≥ 35.0	23,229 (3.45)	2187 (5.01)	4194 (5.60)	708 (2.97)	
Unknown	26,368 (3.92)	785 (1.80)	3424 (4.57)	7097 (29.81)	

### The Association of Smoking With Isolated Proteinuria

Women who quit smoking and those who continued smoking were associated with having a diagnosis of isolated proteinuria both in the unadjusted and adjusted models (Table 2). The adjusted OR for the association between the women who quit smoking and isolated proteinuria was 1.31 (95% CI = 1.14–1.52), and for the women who continued smoking and isolated proteinuria was 1.29 (95% CI = 1.15–1.46). Maternal prepregnancy BMI was found to be a significant predictor for isolated proteinuria.

**Table 2:** The results from the logistic regression model estimating the association between smoking and the risk of isolated proteinuria

Variables	Maternal smoking data			Total
	No smoking	Quit smoking	Continued smoking	
Isolated proteinuria				
No, *n*	641,008	42,458	70,569	754,035
Yes, *n* (per 1000)	1944 (3.0)	207 (4.9)	338 (4.8)	2489 (3.3)
Crude OR (95% CI)	1 (reference)	1.61 (1.39–1.86)	1.57 (1.40–1.76)	
Adjusted OR (95% CI)	1 (reference)	1.31 (1.14–1.52)	1.29 (1.15–1.46)	

CI, confidence interval; OR, odds ratio. Adjusted by maternal age, parity, and prepregnancy body mass index.

### The Association of Smoking and Isolated Proteinuria With Preterm Birth

In the standard comparison, we found evidence of an association between isolated proteinuria (OR = 1.24, 95% CI = 1.03–1.49) in women who quit smoking (OR = 1.09, 95% CI = 1.04–1.15) and women who continued smoking (OR = 1.45, 95% CI = 1.40–1.50) with the risk for preterm birth (Table 3). Thus, we estimated the interaction of SDP and isolated proteinuria, which was found not to be significant (*P* = 0.85, Table 3).

**Table 3:** The results from the logistic regression model estimating the association of smoking and isolated proteinuria with preterm birth

Variables	Preterm birth	Yes, *n* (per 1000)	Standard model	*P*	Interaction model	*P*
	No, *n*		OR (95% CI)		OR (95% CI)	
Maternal smoking						
No	621,789	24,465 (37.9)	ref			<0.0001
Quit	41,194	1711 (39.9)	1.09 (1.04–1.15)	0.0007		
Continue	67,776	3671 (51.4)	1.45 (1.40–1.50)	<0.0001		
Isolated proteinuria						0.036
No	728,383	29,726 (39.2)	ref			
Yes	2376	121 (48.5)	1.23 (1.03–1.49)	0.023		
Interaction of isolated proteinuria and smoking						0.848
Isolated proteinuria × no smoking					ref	
Isolated proteinuria × quit smoking					1.37 (0.74–2.51)	
Isolated proteinuria × continued smoking					1.11 (0.70–1.76)	

CI, confidence interval; OR, odds ratio.

### The Association of Smoking and Isolated Proteinuria With Duration of Pregnancy

We further investigated the association between isolated proteinuria and SDP on duration of pregnancy in pregnancies with gestational age of ≥32 weeks. Duration of pregnancy was shorter in women who had isolated proteinuria (days = −1.54, standard error = 0.20, *P* < 0.0001) compared to other women. Similarly, continued smoking was associated with shorter duration of pregnancy (days = −0.89, SE< 0.01, *P* < 0.001) compared to nonsmoking. On the contrary, women who quit smoking were associated with a longer duration of pregnancy (days = 0.19, standard error = 0.05, *P* = 0.002) compared to nonsmoking. The mean, standard error, and CI for the duration of pregnancy is presented in Table 4.

**Table 4:** The results from the linear regression model estimating the association of smoking and isolated proteinuria with the duration of pregnancy

Variables	Standard model		Interaction model	
	*b* (95% CI)	P	*b* (95% CI)	P
Maternal smoking				
No	ref		ref	
Quit	0.19 (0.07–0.31)	0.006	0.44 (−0.42 to 1.31)	0.45
Continued	−0.89 (−0.99 to −0.80)	<0.0001	−0.88 (−1.58 to −0.18)	0.0087
Isolated proteinuria				
No	ref		ref	
Yes	−1.54 (−1.94 to −1.14)	<0.0001	−1.40 (−2.00 to −0.80)	<0.0001
Maternal age	−0.062 (−0.067 to −0.058)	<0.0001	−0.062 (−0.067 to −0.058)	<0.0001
Parity	−0.30 (−0.32 to −0.28)	<0.0001	−0.30 (−0.32 to −0.28)	<0.0001
BMI				
< 20	−1.13 (−1.23 to −1.04)	<0.0001	−1.13 (−1.23 to −1.04)	<0.0001
20.0–24.9	ref		ref	
25.0–29.9	0.26 (0.18–0.34)	<0.0001	0.26 (0.18–0.34)	<0.0001
30.0–34.9	−0.07 (−0.19 to 0.05)	0.45	−0.07 (−0.19 to 0.05)	0.45
≥ 35.0	−0.58 (−0.75 to −0.42)	<0.0001	−0.58 (−0.75 to −0.42)	<0.0001
Isolated proteinuria × no smoking			ref	
Isolated proteinuria × quit smoking			0.71 (−1.39 to 2.80)	0.93
Isolated proteinuria × continued smoking			−0.87 (−2.56 to 0.83)	0.69

*b*, beta; BMI, body mass index; CI, confidence interval.

Estimates show the impact on pregnancy duration by days.

Estimates for categorical variables are the least squares means differences from the reference category.

*P*-values are Tukey adjusted.

**Figure 1:**
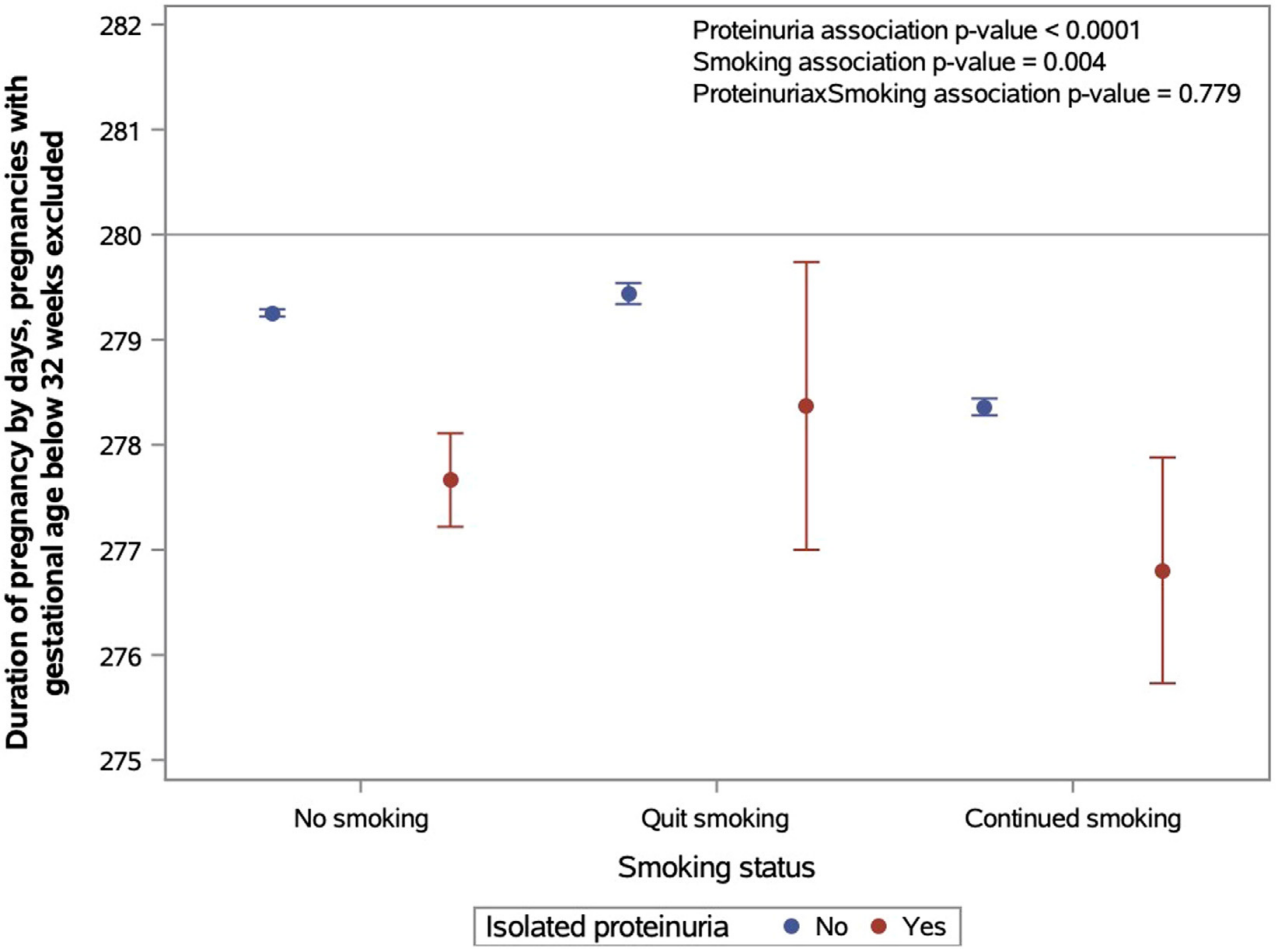
The results from the logistic regression model estimating the interaction of the associations between SDP and isolated proteinuria with the duration of pregnancy in days among offspring born at 32 gestational weeks or more. Gestational age 40 weeks + 0 days = 280 days was used as a reference. SDP, smoking during pregnancy.

The results of the interaction analyses by isolated proteinuria and SDP with the duration of pregnancy are depicted in Figure 1. The interaction between isolated proteinuria and SDP was not statistically significant (*P* = 0.78). Proteinuria was a significant predictor in the model (*P* < 0.0001), such that the duration of pregnancy was shorter in women with proteinuria compared to women without proteinuria. Similarly, SDP was a significant predictor in the model (*P* = 0.004). There was only a trend for shorter duration of pregnancy in women with isolated proteinuria who continued smoking (mean = 276.80, days = −1.56, standard error = 0.55, *P* = 0.052) compared to women who smoked without isolated proteinuria (mean = 278.36).

In the sensitivity analyses, we investigated the association of isolated proteinuria and SDP with the duration of pregnancy in the whole population, including early preterm births. The results showed a similar pattern (Supplementary Figure S1 and Supplementary Table S1).

## Discussion

This is the first study to find evidence of an association between maternal smoking and increased risk for isolated proteinuria during pregnancy, which might be the first sign of smoking-related renal damage in women who smoke. Even though continued SDP seemed to lower the risk for preeclampsia and gestational hypertension,^8^ it seems that smokers have an increased risk for isolated proteinuria instead. Both SDP and isolated proteinuria were associated with a shorter duration of pregnancy and a higher risk of preterm birth among women without previous kidney diseases, gestational diabetes, or preeclampsia. However, the interaction of SDP and isolated proteinuria with preterm birth and duration of pregnancy lacked statistical significance.

Based on our results, the risk of having a diagnosis of isolated proteinuria was increased both among those who continued to smoke and those who quit smoking during the first trimester of pregnancy compared to nonsmokers. This finding contradicts most studies on the harms of smoking, which have shown that smoking throughout pregnancy is more harmful compared to quitting smoking early in pregnancy. Women who smoke generally tend to have other unhealthy lifestyle habits and are more often overweight, which may predispose them to isolated proteinuria compared to nonsmoking women. Smoking and especially nicotine, one of the main harmful ingredients of tobacco, have been shown to have deleterious effects on kidney health through inducing, for example, apoptosis in podocyte cells.^18^, ^19^, ^20^ In addition, smoking has been associated with renal hyperfiltration and albumin leaks across the glomerular filtration barrier.^21^, ^22^, ^23^

We found that isolated proteinuria and continued smoking after the first trimester of pregnancy was associated with a higher risk for preterm birth and shorter duration of pregnancy. These findings are supported by previous studies.^2^^,^^13^ However, we performed interaction analyses of SDP and isolated proteinuria with both preterm birth and duration of pregnancy, but no significant interaction was found.

One previous study with 157 women with proteinuria during pregnancy showed that 34% of them developed preeclampsia.^24^ Those who developed preeclampsia more often had preterm deliveries (at 33 weeks of gestation on average) compared to those who had isolated proteinuria (at 36 weeks of gestation on average). However, 47% of the women with isolated proteinuria had a preterm delivery before 37 weeks of gestation.^24^ It is noteworthy that our study population included healthy women because we excluded women with previous kidney disease, gestational diabetes, or preeclampsia. In our study, isolated proteinuria increases the risk for preterm delivery by 23 %. The reason for the increased risk for preterm delivery may also be explained by the fact that proteinuria could be considered to be part of preeclampsia, and this may precipitate earlier delivery because clinicians may become nervous about continuing pregnancy.

Our findings raise concerns about the renal function of women who smoke during pregnancy. First, there is a need for studies on the combined association of maternal smoking and BMI with kidney function during pregnancy and later. Second, in the future, it should be investigated whether SDP combined with isolated proteinuria increases the risk for later kidney diseases and whether these women have increased risk for preeclampsia in their future pregnancies. Previously, pregnancy complications such as hypertensive disorders, gestational diabetes, and preterm delivery have been associated with a higher risk of subsequent chronic kidney disease in women.^25^^,^^26^ It is also known that prepregnancy chronic kidney disease increases the risk for preeclampsia, cesarean delivery, preterm birth, and a newborn that is small for its gestational age.^27^ The more severe the chronic kidney disease, the greater the risk of adverse pregnancy outcomes.^27^ A study by Kendrick *et al.*^28^ of 778 women with chronic kidney disease with matched healthy controls found that those with kidney disease had 52% increased odds of preterm delivery and 33% increased odds of cesarean delivery compared to a healthy control group.

Our study has several strengths, one of which is the use of the mandatory Finnish health registers, which are shown to be reliable for research purposes.^15^ Our study population included information on all singleton pregnancies between 2006 and 2018. We were able to adjust for a wide range of maternal background factors, including prepregnancy BMI. Furthermore, we excluded women with a prepregnancy diagnosis of chronic hypertension or kidney diseases and women with gestational diabetes or preeclampsia from the analyses. Unfortunately, we had no reliable information on maternal alcohol or illicit drug use.

Our study has limitations which need to be acknowledged in interpreting the data. First, SDP was based on maternal self-reporting, which is known to underestimate the true prevalence of smoking. The register information on SDP has been shown to be consistent with data from a Finnish survey.^29^ The Medical Birth Register does not collect the information of number of cigarettes smoked per day; thus, such information was not available. The data contained information on the duration of SDP, which is a significant strength. Second, we had no information on the actual results of the proteinuria measurements or the timing of the diagnosis because isolated proteinuria was based on the ICD-10 code O12 derived from the Finnish Medical Birth Register in this study. However, it is likely that the most severe cases of proteinuria are diagnosed in the outpatient or the inpatient hospital clinics and included in the birth register data. We did not have information on glomerular filtration rate in the women, which is known to be associated with pregnancy outcomes.^30^ Considering the limitations of our study, there is need for further studies to collect more accurate information on levels of proteinuria. In the future, other potential adverse associations of isolated proteinuria on pregnancy and fetal complications need to be investigated.

In conclusion, we found evidence of an association between SDP and the diagnosis of isolated proteinuria. We speculate that isolated proteinuria may be the first sign of smoking-related renal damage in women who smoke. Furthermore, a diagnosis of isolated proteinuria as well as SDP was associated with a shorter duration of pregnancy and a higher risk for preterm birth among women without previous kidney diseases, gestational diabetes, or preeclampsia.

## Disclosure

All the authors declared no competing interests.

## Supplementary Material

Supplementary File (PDF) **Figure S1.** The results from the logistic regression model estimating the interaction of the associations between smoking during pregnancy and isolated proteinuria on duration of pregnancy in days among the entire study population. **Table S1.** The results from the linear regression model estimating the association of smoking and isolated proteinuria with duration of pregnancy in the whole population.

## Footnotes

Supplementary File (PDF)
**Figure S1.** The results from the logistic regression model estimating the interaction of the associations between smoking during pregnancy and isolated proteinuria on duration of pregnancy in days among the entire study population. Gestational age 40 weeks + 0 days = 280 days was used as a reference. **Table S1.** The results from the linear regression model estimating the association of smoking and isolated proteinuria on duration of pregnancy in the whole population.
