# Supplementary material for: Association Between Maternal Smoking, Isolated Proteinuria During Pregnancy and Preterm Birth: A Finnish Registry Analysis
Source: Kidney Int Rep. 2024 Oct 28;10(1):169–76. doi: 10.1016/j.ekir.2024.10.025 (PMC11725821; doi:10.1016/j.ekir.2024.10.025)
Supplement: Supplementary File (PDF) — Figure S1. The results from the logistic regression model estimating the interaction of the associations between smoking during pregnancy and isolated proteinuria on duration of pregnancy in days among the entire study population. Table S1. The results from the linear regression model estimating the association of smoking and isolated proteinuria with duration of pregnancy in the whole population. [file mmc1.pdf]

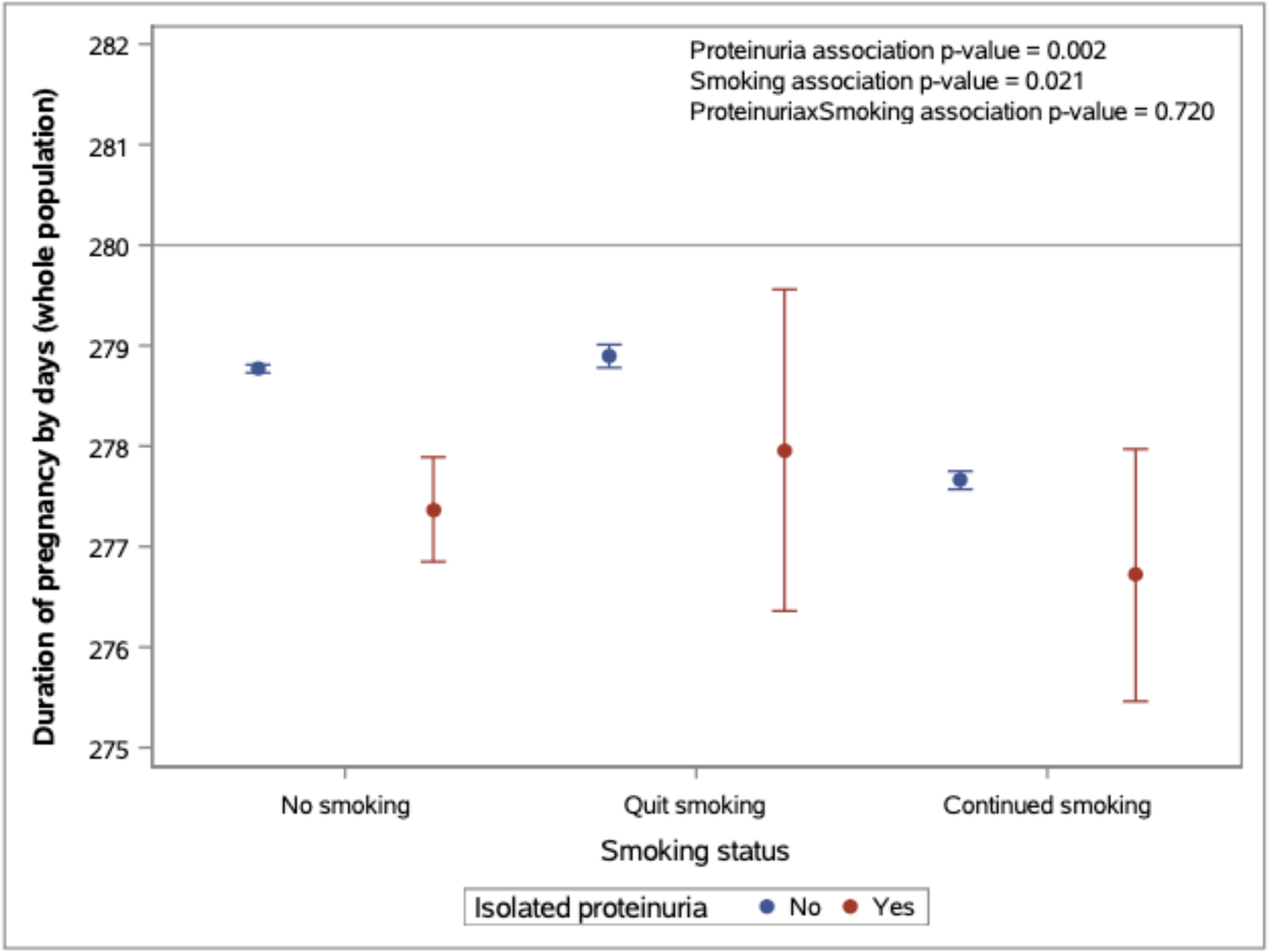

Supplementary Figure S1. The results from the logistic regression model estimating the interaction of the associations between SDP and isolated proteinuria on duration of pregnancy in days among the entire study population. Gestational age 40 weeks + 0 days equaling 280 days was used as a reference.

Supplementary table S1. The results from the linear regression model estimating the association of smoking and isolated proteinuria on duration of pregnancy in the whole population

	Standard model		Interaction model	
	b (95% CI)	*p*	*b (95% CI)*	*p*
Maternal smoking				
No	ref		ref	
Quit	0.13 (-0.01, 0.27)	0.030*	0.36 (-0.65, 1.37)	0.033*
Continued	-1.11 (-1.22, -1.00)	<0.0001*	-0.88 (-1.70, -0.07)	<0.0001*
Isolated proteinuria				
No	ref		ref	
Yes	-1.30 (-1.76, -0.84)	<0.0001	-1.09 (-1.79, -0.39)	<0.0001
Maternal age	-0.076 (-0.081, -0.071)	<0.0001	-0.076 (-0.081, -0.07	<0.0001
Parity	-0.29 (-0.31, -0.27)	<0.0001	-0.29 (-0.31, -0.27)	<0.0001
BMI				
less than 20	-1.21 (-1.33, -1.10)	<0.0001	-1.21 (-1.33, -1.10)	<0.0001
20.0-24.9	ref		ref	
25.0-29.9	0.19 (0.10, 0.29)	<0.0001	0.19 (0.10, 0.29)	<0.0001
30.0-34.9	-0.23 (-0.37, -0.09)	<0.0001	-0.23 (-0.37, -0.09)	<0.0001
35.0 or more	-0.86 (-1.05, -0.67)	<0.0001	-0.86 (-1.05, -0.67)	<0.0001
Isolated proteinuria*No smoking			ref	
Isolated proteinuria*Quit smoking			0.59 (-1.85, 3.03)	0.59*
Isolated proteinuria*Continued smoking			-0.65 (-2.63, 1.33)	0.51*

b , beta; Estimates show the impact on pregnancy duration by days. Estimates for categorical variables are the least squares means differences from the reference category.

*Tukey adjusted p-values
